# Endocarditis-associated rapidly progressive glomerulonephritis mimicking vasculitis: a diagnostic and treatment challenge

**DOI:** 10.1080/07853890.2022.2046288

**Published:** 2022-03-04

**Authors:** Sanxi Ai, Jianzhou Liu, Guotao Ma, Wenling Ye, Rongrong Hu, Shangzhu Zhang, Xiaohong Fan, Bingyan Liu, Qi Miao, Yan Qin, Xuemei Li

**Affiliations:** a Department of Nephrology, Peking Union Medical College Hospital, Chinese Academy of Medical Sciences & Peking Union Medical College, Beijing, China; b Department of Cardiac Surgery, Peking Union Medical College Hospital, Chinese Academy of Medical Sciences & Peking Union Medical College, Beijing, China; c Department of Rheumatology, Peking Union Medical College Hospital, Chinese Academy of Medical Sciences & Peking Union Medical College, Beijing, China

**Keywords:** Infective endocarditis, RPGN, vasculitis, immunosuppressive therapy

## Abstract

**Background:**

Infective endocarditis (IE)-associated rapidly progressive glomerulonephritis (RPGN) is rarely reported. Sporadic case reports have noted the diagnostic and therapeutic challenge in IE-associated glomerulonephritis because it may masquerade as idiopathic vasculitis.

**Methods:**

Patients with clinical diagnosis of IE-related RPGN in a tertiary hospital in China between January 2004 and May 2021 were identified and retrospectively reviewed.

**Results:**

Twenty-four patients with IE-associated RPGN were identified. All patients presented with fever and multiorgan system involvement on top of heart and kidneys, spleen (79%, 19/24), skin (63%, 15/24), lung (33%, 8/24) and nervous system (17%, 4/24). Six of the 24 patients (25%) were initially suspected to have ANCA-associated or IgA vasculitis. Forty-five percent of patients are seropositive for ANCA. Renal histology showed mesangial and/or endocapillary hypercellularity with extensive crescents in most patients. C3-dominant deposition was the predominant pattern on immunofluorescence and pauci-immune necrotising crescentic glomerulonephritis was observed in one case. All patients received antibiotics with or without surgery. Six patients received immunosuppressive therapy before antibiotics due to misdiagnosis and seven patients received immunosuppressive therapy after antibiotics due to persistence of renal failure. Three of the 24 patients died due to severe infection. All the surviving patients had partial or complete recovery of renal function.

**Conclusion:**

IE-associated RPGN is rare and the differential diagnosis from idiopathic vasculitis can be challenging due to overlaps in clinical manifestations, ANCA positivity and absence of typical presentations of IE. The prognosis is generally good if antibiotics and surgery are not delayed. The decision on introducing immunoruppressive treatment should be made carefully on a case by case basis when kidney function does not improve appropriately after proper anti-infective therapy.

**Key messages**
- Infective endocarditis associated RPGN is rare and differentiating it from idiopathic vasculitis can be challenging due to overlap in clinical manifestations, ANCA positivity and occasional absence of typical manifestations of infective endocarditis.
- Kidney function usually responds to antibiotic therapy alone.
- Immunosuppressive therapy may be beneficial in carefully selected patients whose kidney function does not improve with antibiotics alone.

## Introduction

IE often manifests as multi-system involvement due to peripheral septic emboli and/or immunologic phenomena such as cutaneous vasculitis and glomerulonephritis [1], resembling clinical manifestations of idiopathic vasculitis. A substantial proportion (8 ∼ 33%) of patients with IE were positive for ANCA [2–5], which is an useful diagnostic biomarker for ANCA-associated vasculitis. Sporadic case reports have noted that IE-related glomerulonephritis may mimic vasculitis, and it causes a challenge in both diagnosis and treatment [6–9].

IE-associated RPGN is a severe complication of IE, characterized by rapid deterioration of renal function along with features of glomerulonephritis, and it usually presents with crescentic glomerulonephritis (CGN) on renal histology. Due to the rarity of the entity, IE-related RPGN/CGN was reported primarily in case reports. Kannan reviewed IE-related CGN in literature, and only 11 cases were identified before 2000 [10].

The aim of this study is to highlight the diagnostic challenge and discuss the role of immunosuppressive therapy in patients with IE-related RPGN by retrospectively reviewing the clinicopathologic data of such patients in our centre.

## Materials and methods

We retrieved cases with discharge diagnosis including “RPGN” or “CGN” and “IE” from inpatients from January 2004 to May 2021 in Peking Union Medical College Hospital. We also retrieved cases with discharge diagnosis including “acute kidney injury (AKI)” or “acute renal failure” and “IE”. Patients fulfilling the following criteria would be identified as IE-associated RPGN and included in the study: (1) Confirmed diagnosis of IE according to the modified Duke criteria [11]. (2) Diagnosis of RPGN. RPGN is defined as a clinical syndrome characterized by rapid deterioration of renal function accompanied by urinary findings of glomerulonephritis [12]. Clinical diagnosis of RPGN has been made as a diagnosis of exclusion based on rapid decline in kidney function and urinary findings. Clinical diagnosis of RPGN was made if two independent nephrologists proposed RPGN as the most likely diagnosis after comprehensive differential diagnosis, which included pre-renal AKI, post-renal AKI, acute tubular injury, acute interstitial nephritis, renal embolism, and thrombotic microangiopathy. According to kidney biopsy conditions, we further classified patients with clinical diagnosis of RPGN into patients with biopsy-proven CGN (if extensive crescent formation was present on histology) and patients without biopsy-proven CGN (no crescent on histology or without biopsy findings). Patients with KDIGO AKI stage 3 (increase in serum creatinine to 3.0 times baseline or serum creatinine to ≥4.0 mg/dl or the initiation of kidney replacement therapy) were included in the study.

The clinical and pathological data of patients with IE-associated RPGN were retrospectively reviewed. Clinical data were obtained from medical records. Pathological data were reviewed independently by two pathologists. The primary outcome was kidney recovery. For outcome analysis, partial recovery was defined by elevation of serum creatinine >0.2 mg/dl above baseline levels or follow up serum creatinine >1.2 mg/dl (for those in whom baseline levels were unavailable). Complete recovery was defined as elevation of serum creatinine ≤ 0.2 mg/dl baseline or to serum creatinine ≤ 1.2 mg/dl (for those patients in whom baseline levels were unavailable).

This study has been approved by the Institutional Review Board of Peking Union Medical College Hospital (S-K1512).

## Results

### Clinical manifestations

From Jan 2004 to May 2021, 24 patients of IE-related RPGN were identified. The prevalence of IE-related RPGN in IE was estimated to be 3.4% (20 patients with IE-associated RPGN among 595 patients with confirmed IE between June 2012 to May 2021).

The clinical characteristics of the 24 patients are summarized in Table 1. There was a male predominance (3:1) with a mean age of 42 years. Sixty-seven percent (16/24) of patients had pre-existing valvular disease, mainly congenital heart disease and rheumatic valvular disease. No patient was intravenous drug abuser. No patient had past history of chronic kidney disease, idiopathic vasculitis or connective tissue disease. The duration between onset of symptoms to the diagnosis of IE ranged from 1 to 44 weeks with a median disease duration of 14 weeks. All but one patient presented with fever, but 38% (9/24) of cases were afebrile on initial presentations. All patients presented with cardiac murmur and 16 patients developed heart failure. Valvular vegetations were identified on echocardiogram for all patients. Two patients underwent trans-oesophageal echocardiogram due to negative findings on transthoracic echocardiogram. The most commonly involved valve was the aortic valve (12/24), followed by mitral valve (10/24). *Streptococcus viridans* was the most common infectious agents revealed by blood cultures (25%, 6/24), followed by *Staphylococcus aureus* (17%, 4/24), and 38% (9/24) had culture-negative endocarditis.

**Table 1.** Clinical characteristics of IE-related RPGN.

	CGN (*n* = 7)	Without biopsy-proven CGN (*n* = 17)	Total (*n* = 24)
Age (yr)	42 ± 13	42 ± 16	42 ± 15
Male	6 (86%)	12 (70% )	18 (75%)
Hypertension	0 (0%)	5 (29%)	5 (21%)
Diabetes	1 (14% )	1 (6% )	2 (8%)
Prosthetic valve	0 (0%)	2 (12%)	2 (8%)
Multiple valves	1 (14% )	5 (29% )	6 (25%)
Pathogen			
* S.viridans*	2 (29%)	4 (24% )	6 (25%)
* S.aureus*	0 (0%)	4 (24% )	4 (17%)
* Culture negative*	3 (43% )	6 (35% )	9 (38%)
Disease duration (weeks)	13 (IQR 2–25)	12 (IQR 4–16)	12 (IQR 4–22)
System involvement			
* *Heart failure	4 (57% )	12 (71% )	16 (67%)
* *kidney			
* *Gross haematuria	3 (43% )	8 (47% )	11 (46%)
* *Nephrotic syndrome	6 (86% )	9 (53% )	15 (63%)
* *Peak SCr (mg/dl)	6.3 ± 2.2	6.9 ± 3.4	6.7 ± 3.1
* *Skin	4 (57% )	11 (65% )	15 (63%)
* *Purpura	4 (57% )	8 (47% )	12 (50%)
* *Janeway lesion	0 (0%)	3 (18% )	3 (13%)
* *Joints (arthralgia)	2 (29% )	5 (29%)	7 (29%)
* *Spleen	5 (71% )	14 (82% )	19 (79%)
* *Splenomegaly	5 (71% )	14 (82% )	19 (79%)
* *Splenic infarct	1 (14% )	3 (18% )	4 (17%)
* *Lung (embolism)	1 (14% )	7 (41% )	8 (33%)
* *Nervous system (embolism)	1 (14% )	3 (18% )	4 (17%)
* *Eyes (retinal artery embolism)	1 (14% )	1 (6% )	2 (8%)
* *Haematology			
* *Anaemia	7 (100% )	17 (100%)	24 (100%)
* *Thrombocytopenia	3 (43% )	11 (65%)	14 (58%)
* *Pancytopenia	2 (29% )	1 (6%)	3 (13%)
Treatment			
* *Antibiotics	7 (100%)	17 (100%)	24 (100%)
* *Surgery	4 (57%)	13 (76%)	17 (71%)
* *Immunosuppressive therapy	6 (86%)	7 (41%)	13 (54%)
Time of follow up	11 (IQR 8-13)	8 (IQR 3-14)	10.5 (IQR 4-13)
Outcome			
* *Death	1 (14%)	2 (12%)	3 (13%)
* *Renal complete recovery	5 (71%)	9 (53%)	14 (58%)
* *Renal partial recovery	1 (14%)	6 (35%)	7 (29%)

CGN: crescentic glomerulonephritis; RPGN: rapidly progressive glomerulonephritis; *S.viridans: Streptococcus viridans; S.aureus: Staphlococcus aureus*.

All patients presented with rapid deterioration of renal function within 1 week to several months along with glomerular haematuria and proteinuria. The mean peak serum creatinine was 6.7 mg/dl (range 3.0 ∼ 16.1 mg/dl). Fifteen patients underwent haemodialysis when necessary. Seventy-one percent (17/24) of patients had proteinuria ≥3 g/d and 63% (15/24) presented with nephrotic syndrome. Five patients had proteinuria 1.3 ∼ 2.5 g/d and further urine protein electrophoresis suggested mainly glomerular proteinuria (predominantly albuminuria). All the patients showed microhematuria or gross haematuria with many dysmorphic erythrocytes.

All patients had multi-system clinical manifestations other than kidneys and heart, including spleen (79%, 19/24), skin (63%, 15/24), lung (33%, 8/24) and the nervous system (17%, 4/24) (Table 1). It is worth noting that, 25% (6/24) of patients had been initially considered as ANCA-associated vasculitis or IgA vasculitis.

### Laboratory findings

On review of laboratory studies, abnormalities in haematology were common, including anaemia (100%, 24/24), thrombocytopenia (58%, 14/24) and pancytopenia (13%, 3/24) (Table 1). Various markers usually seen in autoimmune diseases were detected with high frequency (Table 2). ANCA was positive in 45% (10/22) of patients, predominantly proteinase 3 (PR3)-ANCA. The titres of PR3-ANCA ranged from 35 to 819 RU/ml with a median titre of 138 RU/ml (reference range <20 RU/ml in our centre). Six patients showed high titres of PR3-ANCA (>100 RU/ml). One patient (case 9) showed dual positivity for PR3-ANCA (>200 RU/ml) and myeloperoxidase (MPO)-ANCA (34 RU/ml). Anti-nuclear antibody was positive in 54% (13/24) of patients, while only one patient showed positive anti-double strand DNA. Hypocomplementemia and elevated rheumatoid factor were presented in 87% (20/23) and 90% (18/20) of patients, respectively.

**Table 2.** Serologic findings in patients with IE-related RPGN.

	CGN (*n* = 7)	Without biopsy-proven CGN (*n* = 17)	Total (*n* = 24)
Hypocomlementemia	5/7 (71%)	15/16 (94%)	20/23 (87%)
Rheumatoid factor	6/6 (100%)	12/14 (86%)	18/20 (90%)
Antinuclear antibody	3/7 (43%)	10/17 (59%)	13/24 (54%)
Coombs’ test	1/3 (33%)	7/9 (78%)	8/12 (67%)
Anti-phospholipid	2/4 (50%)	5/12 (42%)	7/16 (44%)
Type III cryoglobulinemia	3/4 (75%)	3/3 (100%)	6/7 (86%)
c-ANCA	3/7 (43%)	6/15 (40%)	9/22 (41%)
p-ANCA	0/7 (0%)	1/15 (7%)	1/22 (5%)
PR3-ANCA and MPO-ANCA	0/7 (0%)	1/15 (7%)	1/22 (5%)
PR3-ANCA	3/7 (43%)	7/15 (	10/22 (45%)

CGN: crescentic glomerulonephritis; RPGN: rapidly progressive glomerulonephritis; ANCA: antineutrophil cytoplasmic antibodies.

### Pathological findings

**Figure 1.**
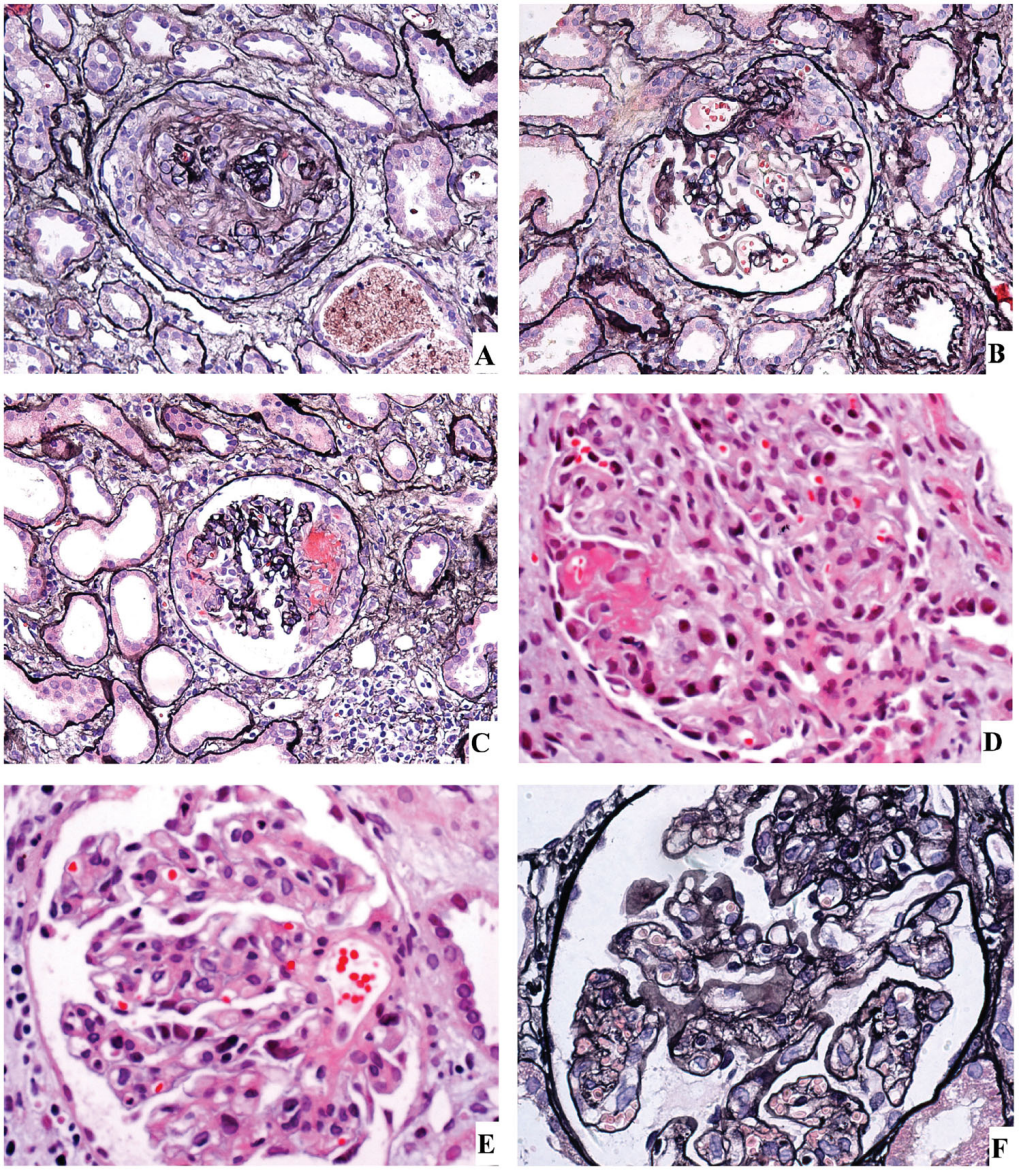
Representative light microscopy findings. (A) Large global cellular crescent (periodic acid-silver methenamine × 200). (B) Small cellular crescent (periodic acid-silver methenamine × 200). (C) Glomerulus with necrotising crescent (periodic acid-silver methenamine × 200). (D) Mesangial and endothelial hypercellularity (haematoxylin and eosin × 400). (E) Mesangial hypercellularity (haematoxylin and eosin × 400). (F) Endocapillary proliferative glomerulonephritis (periodic acid-silver methenamine × 400).

**Figure 2.**
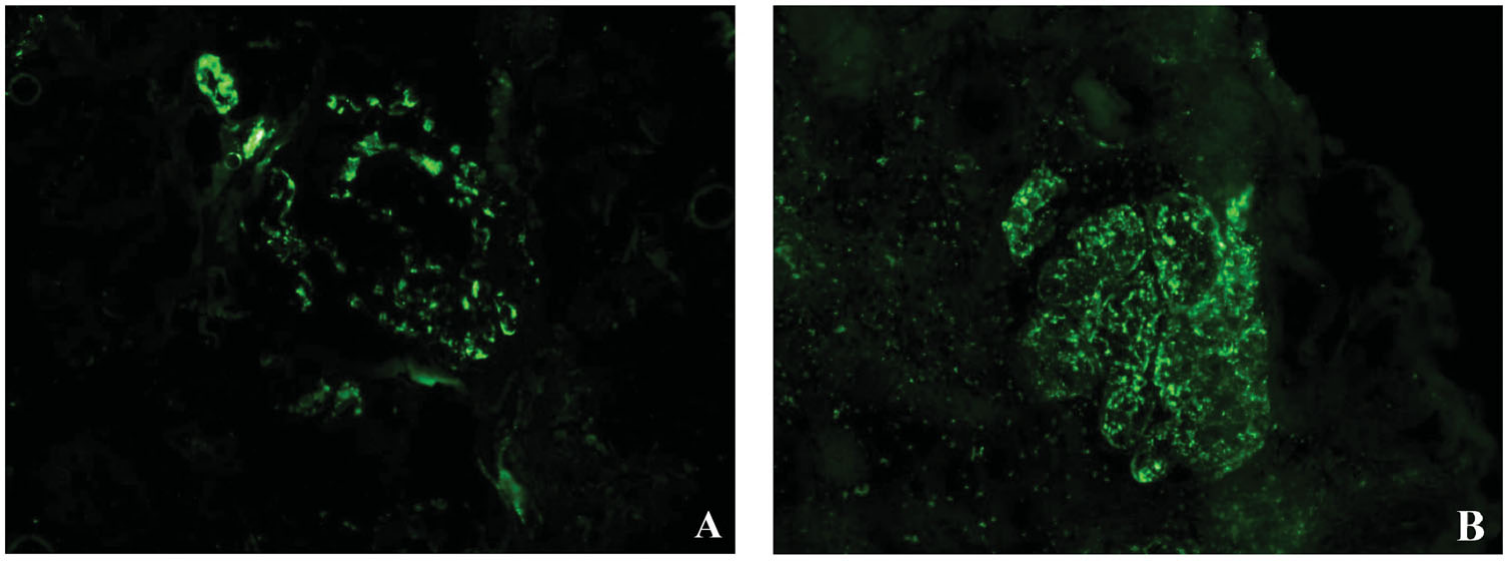
Representative immunofluorescence microscopy findings. (A) Glomerulus with predominantly mesangial staining by C3. (reprinted from Am J Med. 2021;134(12):1539-1545.e1, with permission from Elsevier) (B) Glomerulus with mesangial and capillary wall staining by C3.

**Figure 3.**
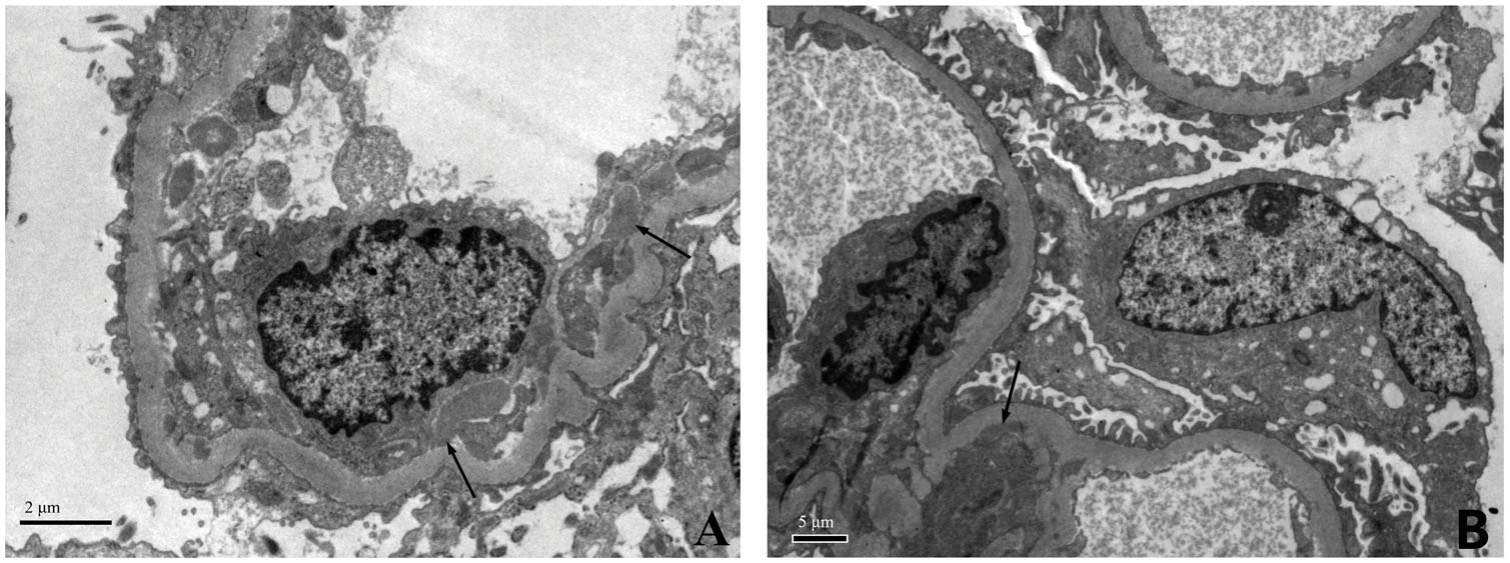
Representative electron microscopy findings. (A) Subendothelial electron-dense deposits (arrow). (B) Mesangial electron-dense deposits (arrow).

Renal biopsies were performed in 9 patients and histologic findings are illustrated in Figures 1–3 and summarized in Table 3. On light microscopy, all patients presented with mesangial and/or endothelial hypercellularity. Extensive crescents formation (accounting for 34 ∼ 67% of glomeruli) were observed in seven of the nine cases. Two cases had clinical presentations of RPGN but no crescent formation on histology. Case 21 presented with focal mesangial hypercellularity and she underwent renal biopsy after two months of treatment when her serum creatinine had decreased from 11.6 mg/dl to 0.8 mg/dl. Case 22 presented with diffuse endocapillary proliferative glomerulonephritis (Figure 1(F)). Fibrinoid necrosis was observed in two of the three cases who were seropositive for ANCA and none of the five cases with negative ANCA. Acute tubular injury and interstitial inflammation were present in all cases. On immunofluorescence, C3 was present in seven of the nine cases and predominant in five. IgM was found in five cases, IgA in three cases, and IgG in one case. One case presented with pauci-immune glomerulonephritis with no significant deposit on immunofluorescence or electronic microscopy. Electron microscopy examination was performed in five cases. Deposits in mesangial and/or subendothelial area were noted in four of the five cases, but subepithelial deposits were occasionally observed in only one case.

**Table 3.** Renal biopsy findings of nine cases.

No.	No. of glomeruli	Light microscopy		Immunofluorescence	Electronic microscopy	Pathogen/ ANCA
		Glomeruli	Tubule and interstitium			
1	23	15 cellular or fibrocelluar crescents. Segmental mesangial hypercellularity	Mild tubular atrophy and interstitial fibrosis	Negative	Mesangial, subendothelial deposit	*S.viridans / +*
2	24	14 cellular or fibrocellular crescents with segmental fibrinoid necrosis. Diffuse mesangial hypercellularity	Mild interstitial fibrosis	Mesangial and capillary loops: IgM 1+, C3 1+	Mesangial deposit	- / +
3	32	7 glomeruli with global sclerosis and 1 with segmental sclerosis (crescent); 16 celluar or fibrocellular crescents with many fibrinoid necrotising areas. Segmental mesangial hypercellularity	Mild tubular atrophy and interstitial fibrosis; Hyalinisation of arterioles	Negative	No deposit	- / +
4	17	6 cellular crescents. Segmental mesangial hypercellularity	Mild tubular atrophy and interstitial fibrosis	Mesangial and capillary loops: IgA 2+, IgM 1+, C3 2+, C1q 1+	NA	*S.viridans / -*
8	5	3 cellular crescents. Diffuse mesangial and segmental endothelial hypercellularity	No tubular atrophy or interstitial fibrosis	Mesangial and capillary loops: IgG +/-, IgM 1+, C3 3+, C1q 1+	NA	- / -
14	18	10 cellular or fibrocellular crescents. Diffuse mesangial and segmental endothelial hypercellularity	NA	Mesangial and capillary loops: IgA +/-, IgM 2+, C3 2+, C1q 2+	NA	*E.coli* / -
17	29	10 cellular or fibrocellular crescents. Diffuse mesangial and endothelial hypercellularity	Mild tubular atrophy and interstitial fibrosis	Mesangial: IgA 3+, C3 3+, C1q 2+, κ 2+, λ 2+	Mesangial, subendothelial deposit	*Gemella* / -
21	12	Focal mesangial hypercellularity	No tubular atrophy or interstitial fibrosis	Mesangial and capillary loops: IgG 2+, IgA 2+, IgM 2+, C3 1+	NA	- / NA
22	25	8 glomeruli with global ischaemic sclerosis; Diffuse endothelial and segmental mesangial hypercellularity	Focal tubular atrophy and interstitial fibrosis; benign arteriolar nephrosclerosis	Mesangial and capillary loops: C3 2+,κ 1+, λ 1+	Mesangial and subendothelial deposit, occasionally subepithelial deposit	*C.striatum* / -

ANCA: antineutrophil cytoplasmic antibodies; NA: not available; *S.viridans*: *Streptococcus viridans*; *E.coli*: *Escherichia coli*; *C. striatum*: *Corynebacterium Striatum*.

### Treatment and outcome

Treatment and follow-up data are presented in Supplemental Table 1 and summarized in Table 4. The median time of follow-up was nine (range 1 ∼ 40) months. All the patients received antibiotics. Seventeen patients had cardiac surgeries (5 ∼ 64 days after antibiotics), 13 of which were performed within 4 weeks after commencing antibiotics. Thirteen patients received immunosuppressive therapy including glucocorticoid with or without cyclophosphamide. Three of the 24 patients died due to severe infection. The remaining 21 patients recovered uneventfully (NHYA class I–II and dialysis independent) during follow up.

**Table 4.** Outcome in patients with IE-related RPGN with or without IS.

	CGN (*n* = 7)		Without biopsy-proven CGN (*n* = 17)		Total (*n* = 24)	
	IS + (*n* = 6)	IS - (*n* = 1)	IS + (*n* = 7)	IS - (*n* = 10)	IS + (*n* = 13)	IS - (*n* = 11)
Age (yr)	42 ± 14	45	38 ± 18	44 ± 15	40 ± 16	44 ± 14
Male	5/6	1/1	4/7	8/10	9/13 (69%)	9/11 (82%)
Comorbidity	0/6	1/1	1/7	4/10	1/13 (8%)	5/11 (45%)
S.viridans	2/6	0/1	1/7	3/10	3/13 (23%)	3/11 (27%)
S.aureus	0/6	0/1	2/7	2/10	2/13 (15%)	2/11 (18%)
Heart failure	3/6	1/1	4/7	8/10	7/13 (54%)	9/11 (82%)
Disease duration (weeks)	10.5 (IQR 2-29)	24	12 (IQR 3-16)	8.5 (IQR 4-18)	12 (IQR 3-21)	12 (IQR 4-24)
Peak SCr (mg/dl)	6.6 ± 2.2	4.1	8.5 ± 4.0	5.9 ± 2.6	7.6 ± 3.3	5.7 ± 2.6
ANCA positivity	2/6	1/1	4/6	4/9	6/12 (50%)	5/10 (50%)
Surgery	3/6	1/1	4/7	9/10	7/13 (54%)	10/11 (91%)
Dialysis	5/6	0/1	3/7	7/10	8/13 (62%)	7/11 (64%)
Time of follow up (months)	11 (IQR 10-14)	2	10 (IQR 4-12)	6.5 (IQR 2-16)	11 (IQR 7-13)	5 (IQR 2-15)
Death	1/6	0/1	1/7	1/10	2/13 (15.5%)	1/11 (9%)
Renal complete recovery	4/6	1/1	5/7	4/10	9/13 (69%)	5/11 (45.5%)
Renal partial recovery	1/6	0/1	1/7	5/10	2/13 (15.5%)	5/11 (45.5%)

IS: immunosuppressive therapy; CGN: crescentic glomerulonephritis; RPGN: rapidly progressive glomerulonephritis; *S.viridans*: *Streptococcus viridans; S.aureus*: *Staphlococcus aureus;* SCr: serum creatinine; ANCA antineutrophil cytoplasmic antibodies.

**Figure 4.**
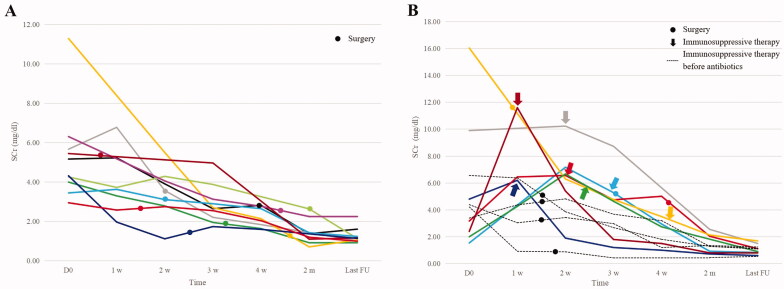
Changes of serum creatinine (SCr) in patients with rapidly progressive glomerulonephritis. D0 indicates the day of commencing antibiotics. Last FU indicates the last follow up. (A) Changes of SCr in 10 surviving patients without immunosuppressive therapy. (B) Changes of SCr in 11 surviving patients with immunosuppressive therapy.

The clinical characteristics and outcome of patients with or without immunosuppressive treatment were summarized in Table 4. Among the 11 patients who did not have immunosuppressive treatment, one patient died due to severe infection. The remaining 10 patients achieved complete (5/10) or partial renal recovery (5/10) after antibiotics and surgery (Figure 4(A)).

Among the 13 patients receiving immunosuppressive therapy, glucocorticoid was added after implementing antibiotics in seven patients with a time interval of 4 ∼ 35 days due to deterioration or no significant improvement of renal function during the course of anti-infective therapy (Figure 4(B)). For the seven patients above, infection was in control and blood cultures were repeatedly negative at the time of commencing immunosuppressive therapy. Five of the seven patients had complete recovery of renal function and two patients had partial recovery. While, six of the 13 patients received glucocorticoid before antibiotics due to delayed diagnosis of IE. Two of the six patients died due to severe infection. The two patients had received immunosuppressive therapy for two or three months before the diagnosis of IE, and they both developed pneumonia and heart failure during this period. The remaining four patients survived and had complete renal recovery after anti-infective treatment (Figure 4(B)). Three of them had received immunosuppressive therapy for six days to one month before the diagnosis of IE and they developed severe adverse events (cerebral embolism, new onset or deterioration of heart failure, respectively) during this period. One patient received antibiotic after glucocorticoid on the same day due to timely corrected diagnosis, and no severe adverse event occurred during immunosuppression.

## Discussion

IE-associated RPGN is rare and was primarily described in case reports [6–9], but the exact prevalence of RPGN in IE is unknown. For the first time, we evaluated the prevalence of IE-associated RPGN, which accounted for 3.4% (20/595) of cases with IE in our centre. The study presented a retrospective observational case series of IE-related RPGN. 24 cases were identified, and 25% (6/24) were initially considered as idiopathic vasculitis (ANCA-associated vasculitis or IgA vasculitis), highlighting the diagnostic challenge. Differentiating IE-related RPGN from idiopathic vasculitis is of obvious importance, given the totally different therapeutic strategies of the two entities.

IE may mimic idiopathic vasculitis partly due to overlaps in clinical manifestations between the two entities. ANCA-associated vasculitis is a form of small-vessel vasculitis associated with ANCA, with predominant involvement of the upper and lower respiratory tract, kidney, skin and the nervous system. IE may closely mimic the clinical manifestations of ANCA-associated vasculitis, by presenting with similar clinical manifestations, including constitutional symptoms (fever, weight loss), involvement of skin, kidney, lung and the nervous system. Zhang et al. reviewed 27 cases of ANCA positive IE-related glomerulonephritis in literature, and involvement of skin, lung and the nervous system were present in 22.2%, 18.5% and 14.8% of patients respectively [6]. In our cohort of IE-related RPGN, involvement of skin, pulmonary and the nervous system were observed in 63%, 33% and 17% of patients respectively. IgA vasculitis is another form of small-vessel vasculitis characterised by IgA-dominant immune deposits. IE mimicking IgA vasculitis by manifesting as glomerulonephritis and purpura was rarely reported [9]. In our study, 50% (12/24) of patients with IE-related RPGN presented with purpura, overlapping with the manifestations of IgA vasculitis.

The presence of ANCA in IE further complicates the differential diagnosis between IE and ANCA-associated vasculitis. ANCA directed against PR3 or MPO are well-known diagnostic markers for ANCA-associated vasculitis. However, ANCA positivity has been reported in various chronic infections including IE. Several studies showed that ANCA (mostly PR3) can be found in IE with a prevalence of 8 ∼ 33% by ELISA [2–5], some with high titres. Bartonella endocarditis may have higher prevalence (40%∼67% by ELISA) [13,14]. In the present study, 45% (10/22) of patients with IE-associated RPGN had ANCA (by ELISA) positivity, consistent with previous reports. The presence of ANCA in addition to vasculitis-like clinical manifestations in patients with IE may easily lead to an erroneous diagnosis of idiopathic vasculitis. Some of the proposed mechanisms of ANCA formation during infectious episodes include autoantigen complementarity, molecular mimicry, neutrophil extracellular traps dysfunction, Toll-like receptors and epigenetics [15]. It is believed that ANCA is pathogenic in ANCA-associated vasculitis [16]. The pathogenic role of ANCA in IE is unclear. But Langlois et al. suggested that ANCA may be linked with more frequent renal impairment and multiple valve involvement in IE [4].

Despite the overlapping clinical manifestations and ANCA positivity in IE, distinctions do exist between ANCA-positive IE and ANCA-associated vasculitis. Previous studies revealed that a high frequency of fever, constitutional symptoms, renal involvement and skin lesions was displayed in both conditions. However, cardiac murmur, splenomegaly, thrombocytopenia, hypocomplementemia and other auto-antibodies were predominantly associated with IE [17–19]. The differentiation between IE-associated glomerulonephritis and purpura from IgA vasculitis should not be difficult if differential diagnosis is considered. The presence of fever together with cardiac murmur favour the diagnosis of IE over IgA vasculitis. In our study, fever, cardiac murmur and splenomegaly were presented in 96%, 100% and 79% of patients, respectively, with high frequencies of thrombocytopenia, hypocomplementemia and multiple auto-antibodies, thus favouring a diagnosis of IE over vasculitis. More importantly, positive blood cultures and valvular vegetation on echocardiography strongly support the diagnosis of IE. But these investigations may be delayed when patients are afebrile. In our study, 38% (9/24) of patients were afebrile on initial presentations, and investigations for IE were delayed in these patients. It is also important to take into consideration that patients with IE may have negative findings on blood cultures or transthoracic echocardiography [20,21], which occurred in 38% (9/24) and 8% (2/24) of patients in our study.

Although renal biopsy is not a routine examinations in patients with IE-associated glomerulonephritis. In patients with elusive diagnosis, histologic findings can provide additional clues for diagnosis, but overlaps exist between IE-related glomerulonephritis and idiopathic vasculitis. The typical renal histology of ANCA-associated vasculitis is pauci-immune necrotizing CGN without endocapillary hypercellularity [22]. On the contrary, IE-related glomerulonephritis typically presented with endocapillary and/or mesangial hypercellularity with frequent crescent formation and C3-dominant immune deposits on immunofluorescence [5,10]. But pauci-immune necrotizing CGN might be rarely observed in IE [6,8,23], regardless of ANCA positivity. Consistent with previous studies, all the 9 cases in our study presented with mesangial and/or endocapillary hypercellularity with extensive crescents formation in most patients. All but one patient had immune deposits with a C3-dominant pattern in most patients. One patient displayed pauci-immune necrotizing CGN and positive ANCA in serum, closely mimicking ANCA-associated vasculitis. But prominent mesangial hypercellularity was observed in this patient, thus favouring the diagnosis of infection over ANCA-associated vasculitis. Taken together, although pauci-immune necrotizing CGN can be rarely observed in IE-related glomerulonephritis, immune deposits and endocapillary proliferation usually favour a diagnosis of infection over ANCA-associated vasculitis. Histologic features of IE-related glomerulonephritis may resemble IgA vasculitis by presenting as mesangial hypercellularity with IgA-dominant immune deposits. Stronger staining of C3 than IgA on immunofluorescence and subepithelial humps on electronic microscopy favours the diagnosis of IE over IgA vasculitis [24].

In terms of treatment, prompt and effective antibiotics is the cornerstone of treatment for IE. Cardiac surgery may also be necessary in certain conditions such as severe heart failure, and there has been a trend preferring earlier surgery [25]. The role of immunosuppressive treatment is controversial in IE-associated RPGN/CGN because of the possible risk of aggravating infection and resolution of the renal disease by antibiotics only in some patients [26,27]. While there are also some case reports showing that antibiotics alone were not effective and only combination therapy of glucocorticoid and antibiotics rescued renal function [8,23]. In previous literature reviews, about 50% of cases with IE-associated RPGN received immunosuppressive therapy and benefit of immunosuppressive therapy was suggested [6,10]. In our study, 10 of the 11 patients without immunosuppressive therapy had complete or partial renal recovery, suggesting good renal prognosis after effective anti-infective treatment in these patients. Nevertheless, seven patients in our study received immunosuppressive therapy due to persistence of renal failure during the course of anti-infective treatment. And their renal function improved after the addition of immunosuppressive therapy (Figure 4(B)), suggesting the potential benefits of immunosuppressive therapy. A higher rate of complete renal recovery was observed in patients with immunosuppressive therapy compared to patients without (69% vs 45%). But the comparison was limited by the non-balanced distribution of confounding factors such as surgery and comorbidities (Table 4) and the small sample size. Therefore, no conclusion could be drawn based on the comparison. Immunosuppressive therapy may be individualised after careful evaluation of risk and benefit. Some suggested to combine antibiotics and immunosuppressive agents when renal failure does not improve after antibiotics within a proper period of time [28,29]. However, immunosuppressive treatment should not be implemented before effective anti-infective therapy. In our study, two of the six patients who received glucocorticoid before antibiotics died, while all of the seven patients who received glucocorticoid after antibiotics survived, suggesting the importance of the timing of immunosuppressive therapy.

There are several limitations in the study. First, the sample size is relatively small. Studies with larger sample size are warranted in the future to evaluate the role of immunosuppressive therapy in IE-associated RPGN. Secondly, only nine of the 24 patients underwent kidney biopsy. For the remaining 15 patients without kidney biopsy, CGN was not confirmed. Finally, this is a retrospective study with various time of follow-up. Prospective studies with pre-specified follow-up are warranted in the future.

## Conclusions

In summary, the study suggests that IE-related RPGN is a rare complication of IE and it may mimic idiopathic vasculitis due to overlaps in clinical and histological features between the two entities and ANCA positivity. Clinicians should be aware of the diagnostic pitfall to avoid erroneous diagnosis and harmful treatment decisions. Renal prognosis is generally good after effective anti-infective treatment. Immunosuppressive therapy may be considered carefully on a case by case basis when kidney function does not improve appropriately after proper anti-infective therapy.

## Supplementary Material

Supplemental Material

## Data Availability

The data during the current study are available from the corresponding author on reasonable request.
