# Supplementary material for: Endocarditis-associated rapidly progressive glomerulonephritis mimicking vasculitis: a diagnostic and treatment challenge
Source: Ann Med. 2022 Mar 4;54(1):754–63. doi: 10.1080/07853890.2022.2046288 (PMC8903796; doi:10.1080/07853890.2022.2046288)
Supplement: Supplemental Material [file IANN_A_2046288_SM0451.docx]

Supplemental Table 1. Treatment and outcome of patients with IE-related RPGN

| No. | Initial doses of steroids | CTX | Timing / Duration of IS | Abx / Surgery | HD | ANCA | Baseline Scr (mg/dl) | Peak SCr (mg/dl) | Scr on FU (mg/dl) | Out-  come | Time of FU |
| --- | --- | --- | --- | --- | --- | --- | --- | --- | --- | --- | --- |
| Crescentic glomerulonephritis (n=7) | | | | | | | | | | | |
| 3 | - | - | - | + / + | - | + | 0.9 | 4.1 | 0.9 | CR | 2 m |
| 1 | Pulse → MP 40mg/d | 0.2g iv qod total 12g | 4 days post-Abx / 8 m | + / - | - | + | normal | 6.2 | 0.6 | CR | 13 m |
| 2 | Pulse → MP 68mg/d | 0.05g po qd  total 12g | 15 days post-Abx / 6 m | + / - | + | + | NA | 9.9 | 1.5 | PR | 11 m |
| 4 | Pulse → Pred 60mg/d | 0.4g iv twice | 1 month pre-Abx / 12 m | + / + | + | - | 1.0 | 4.4 | 1.2 | CR | 11 m |
| 8 | Pulse → Pred 60mg/d | - | 15 days post-Abx / 6 m | + / + | - | - | NA | 6.6 | 1.1 | CR | 16 m |
| 17 | MP 80mg/d | 0.6g iv once | 1month pre-Abx / 12 m | + / + | + | - | NA | 4.4 | 1.0 | CR | 11 m |
| 14 | Pred 60mg/d | 0.1g po qd, total 9.0g | 3 months pre-Abx / 4 m | + / - | + | - | 0.6 | 8.3 | died | died | 8 m |
| Clinical RPGN (n=17) | | | | | | | | | | | |
| 5 | - | - | - | + / + | + | - | 1.1 | 5.7 | 1.6 | PR | 15 m |
| 7 | - | - | - | + / + | - | - | 1.0 | 9.5 | 1.0 | CR | 5 m |
| 9 | - | - | - | + / + | - | + | 0.6 | 11.3 | 1.1 | PR | 20 m |
| 11 | - | - | - | + / + | - | + | NA | 4.3 | 1.1 | CR | 13 m |
| 12 | - | - | - | + / + | - | + | NA | 6.3 | 2.3 | PR | 2 m |
| 15 | - | - | - | + / + | + | - | NA | 3.6 | 1.3 | PR | 36 m |
| 16 | - | - | - | + / + | + | - | 0.8 | 5.9 | 1.2 | PR | 4 m |
| 18 | - | - | - | + / + | - | - | NA | 4.3 | 1.1 | CR | 2 m |
| 20 | - | - | - | + / - | + | - | NA | 4.7 | died | died | 1.5 m |
| 23 | - | - | - | + / + | + | + | NA | 3.0 | 1.1 | CR | 8 m |
| 6 | MP 80mg/d | - | 21 days  post-Abx / 8 m | + / + | - | + | normal | 7.2 | 0.8 | CR | 40 m |
| 10 | Pred 60mg/d | - | 35 days  post-Abx / 6 m | + / + | + | + | 1.2 | 16.1 | 1.7 | PR | 10 m |
| 19 | MP 40mg/d | - | 17 days  post-Abx / 1m | + / - | - | + | 0.7 | 6.7 | 0.9 | CR | 12 m |
| 21 | Pulse →  Pred 50mg/d | - | 9 days post-Abx / 6 m | + / - | - | - | 0.6 | 11.6 | 0.8 | CR | 6 m |
| 13 | MP 40mg/d | - | same day / 2 m | + / + | - | - | 0.5 | 4.2 | 0.5 | CR | 12 m |
| 24 | MP 80mg/d | - | 6 days  pre-Abx / 4 m | + / + | + | + | 1.1 | 6.6 | 1.1 | CR | 4 m |
| 22 | MP 80mg/d | - | 2 months pre-Abx / 3 m | + / - | + | - | NA | 7.1 | died | died | 1 m |

RPGN rapidly progressive glomerulonephritis; CTX cyclophosphamide; IS immunosuppressive therapy; Abx antibiotics; HD hemodialysis; ANCA antineutrophil cytoplasmic antibodies; FU follow up; Pulse methylprednisolone 500-1000 mg/d for 3 days; MP methylprednisolone; Pred prednisone; SCr serum creatinine; CR complete renal recovery; PR partial renal recovery; NA not available, m month.
